# Shining a Light on Intestinal Traffic

**DOI:** 10.1155/2012/808157

**Published:** 2011-11-22

**Authors:** Carola T. Murphy, Kenneth Nally, Fergus Shanahan, Silvia Melgar

**Affiliations:** Alimentary Pharmabiotic Centre, University College Cork, National University of Ireland, Cork, Ireland

## Abstract

Inflammatory bowel disease (IBD), encompassing Crohn's disease and ulcerative colitis, is associated with enhanced leukocyte infiltration to the gut, which is directly linked to the clinical aspects of these disorders. Thus, leukocyte trafficking is a major target for IBD therapy. Past and emerging techniques to study leukocyte trafficking both *in vitro* and *in vivo* have expanded our knowledge of the leukocyte migration process and the role of inhibitors. Various strategies have been employed to target chemokine- and integrin-ligand interactions within the multistep adhesion cascade and the S1P/S1PR1 axis in leukocyte migration. Though there is an abundance of preclinical data demonstrating efficacy of leukocyte trafficking inhibitors, many have yet to be confirmed in clinical studies. Vigilance for toxicity and further research is required into this complex and emerging area of IBD therapy.

## 1. Introduction

Leukocyte migration is fundamental to immunologic mobilization in response to insult and injury. A coordinated series of molecular events underpins the trafficking of lymphocytes and granulocytes into stressed tissue with participation of adhesion molecules, chemokines, and their receptors.

The inflammatory bowel diseases (IBDs), Crohn's disease (CD) and ulcerative colitis (UC), are distinct syndromes, but both are associated with enhanced leukocyte trafficking to the inflamed gut. Thus, targeting the multistep leukocyte adhesion cascade has been employed as a therapeutic strategy. Here, we focus on the contribution of leukocyte trafficking to the pathogenesis of IBD. An overview of strategies employed to target leukocyte recruitment and of emerging models used to test these targets is presented.

## 2. IBD

Crohn's disease and ulcerative colitis are recurring, relapsing, and remitting disorders characterized by chronic inflammation of the intestinal mucosa. Though they share some common clinical symptoms such as diarrhea and abdominal pain, CD and UC possess very distinct features. Crohn's disease manifests as a transmural inflammation that can potentially develop anywhere along the gastrointestinal tract but primarily occurs in the terminal ileum and proximal colon. Ulcerations, granulomas, and bowel fistulas are characteristic histopathological features. In contrast, ulcerative colitis seldom ulcerates and is a relatively superficial inflammation of the mucosa that is diffuse, continuous, and restricted to the colon, usually extending proximally from the rectum. It is characterized by significant goblet cell depletion and crypt abscesses, but granuloma development is not a feature. In both CD and UC, leukocyte infiltration into the inflamed intestine is fundamental to disease development and perpetuation. The infiltrated effector cells resist apoptosis and persistently release harmful inflammatory cytokines causing tissue damage. In CD, the characteristic granulomas form upon dense accumulation of activated T cells and macrophages. Ulcerative colitis is characterized by excessive mucosal infiltration of T cells and neutrophils, the latter forming the characteristic crypt abscesses [1]. The pivotal role of T cells, neutrophils, and their proinflammatory cytokines in the pathogenesis of IBD has been reviewed elsewhere [2, 3]. 

Genetic and environmental risk factors have been implicated in IBD (for reviews see Xavier and Podolsky [1], Melgar and Shanahan [4], and Cho and Brant [5]). Despite extensive research, current therapeutic options in IBD remain limited, often varying in their maintenance, toxicity, and tolerability [6–8]. Novel therapeutic strategies for IBD are needed. 

## 3. Leukocyte Trafficking

### 3.1. *In Vitro* and *In Vivo* Models of Leukocyte Trafficking

#### 3.1.1. Traditional Methods

The concept of lymphocyte recirculation and homing was first demonstrated by Gowans and Knight in 1964, when they followed the migration of radiolabeled lymphocytes in rats using autoradiography and scintography [9]. They and others found that while naïve lymphocytes migrated to all secondary lymphoid tissues, activated lymphocytes preferentially migrated back to the tissue in which they had been exposed to the antigen. In order to devise rational therapeutic strategies that target leukocyte migration, it is important to elucidate leukocyte trafficking patterns *in vivo. *Techniques employed to study leukocyte trafficking frequently involve *ex vivo *labeling of donor cells and adoptive transfer into recipient animals. Subsequently, the distribution of the labeled cell population in recipient tissues is assessed using a variety of imaging methods, such as histological analysis of fixed tissues. This technique has provided us with valuable mechanistic knowledge, but it does not permit direct examination of dynamic processes at single-cell level or provide temporal or spatial information within the physiological environment of lymphoid tissues. Furthermore, labeling of cells *ex vivo *has been associated with variable labeling efficiency, alteration of cellular functions, and label elution postadoptive transfer. Radiolabeling techniques, such as white cell scintigraphy, have been used to successfully study neutrophil migration in clinical IBD studies [10]. However, such methods are hampered by radioactive decay, poor resolution, and cellular toxicity [11]. Myeloperoxidase (MPO) assays are commonly used to study neutrophils and quantitate neutrophil influx, but they do not distinguish between neutrophils and macrophages and can be problematic to carry out. Similarly, while* in vitro* chemotaxis assays are regularly employed to analyze the effects of potential inhibitors on cell migration, there is no guarantee that the cells will respond in the same way to the test compound *in vivo.* Multiphoton intravital microscopy has been widely used to image the dynamic movement of lymphocytes by tracking fluorescently labeled cells in exposed or explanted lymph nodes (LNs) of living animals. This technique has provided valuable insights into the dynamics of T- and B-cell homing to LNs [12, 13] and allows single-cell tracking, in conjunction with high-resolution images. Nonetheless, intravital microscopy is an invasive technique, and the surgery required may interfere with the flow of blood and lymph creating experimental artifacts.

#### 3.1.2. Molecular Imaging Techniques

Molecular imaging is defined as ‘‘the visualization, characterization and measurement of biological processes at the molecular and cellular levels in humans and other living systems” [14]. Over the past decade, a number of such techniques have been adapted to small animal imaging, offering dynamic imaging methods to localize leukocytes *in vivo*. Positron emission tomography (PET) [15] and single-photon emission-computed tomography (SPECT) [16, 17] use scintillation cameras and other devices to detect radioactive emission from radiolabeled cells within the body. Though these nuclear imaging methods have excellent tissue penetration and cell quantitation capability, they too are subject to the drawbacks associated with radiotracers and exogenous labeling of cells. Magnetic resonance imaging (MRI), which relies on the nuclear resonance of protons in tissues upon scanning with radio frequency radiation, has shown great promise to track the recruitment of, for example, antigen-specific CD8^+^ T cells to target tumors *in vivo *[18]. However, the time required for imaging using this technique makes it unsuitable for tracking fast-moving cells [19]. The substantial expense of MRI and its relatively poor sensitivity and quantitative capability have also hindered its use as a basic research tool. Optical fluorescence imaging has been frequently used to track T cells and has provided us with invaluable data on their migration patterns. The pitfalls with using fluorescent labels, such as CFSE and GFP, include signal loss due to label dilution upon cell division and limited sensitivity and specificity due to endogenous tissue autofluorescence and light scattering and absorption [20].

#### 3.1.3. Bioluminescence Imaging

To illuminate cell trafficking *in vivo* and to test specific inhibitors of this migration, we used bioluminescence imaging (BLI) technology [21–23]. This form of optical imaging has several advantages over other techniques for tracking cell migration. It eliminates the necessity to prelabel cells, avoiding problems with exogenous cell labeling. It allows direct *in vivo* and *ex vivo* visualization, with no further processing of tissues required, and is, therefore, a less complicated and less labor intensive technique than most other *in vitro/ex vivo *methods. Unlike other molecular imaging methods, BLI combines high sensitivity with relatively low cost while providing quantitative, spatial, and temporal data. However, this technology has limitations. Since light transmission through animal tissues is wavelength dependent, loss of photon signal can occur with tissue depth and light sources closer to the surface of the animal can appear brighter. In addition, validation of potential therapeutics using BLI should be carried out in conjunction with *in vitro* mechanistic assays and *in vivo* efficacy studies. BLI has successfully monitored trafficking of bone marrow mononuclear cells in ischemic myocardium [24] and CD4^+^ T cells in an experimental model of multiple sclerosis (MS) [25]. These and other studies have shown robust and reliable correlation between cell numbers and bioluminescence signals. We employed BLI to track both neutrophils and lymphocytes in murine models of experimental colitis and to test potential inhibitors of their migration Figure 2 [21, 22].

### 3.2. The Leukocyte Trafficking Cascade

#### 3.2.1. Selectins, Integrins, and Their Ligands

**Figure 1:**
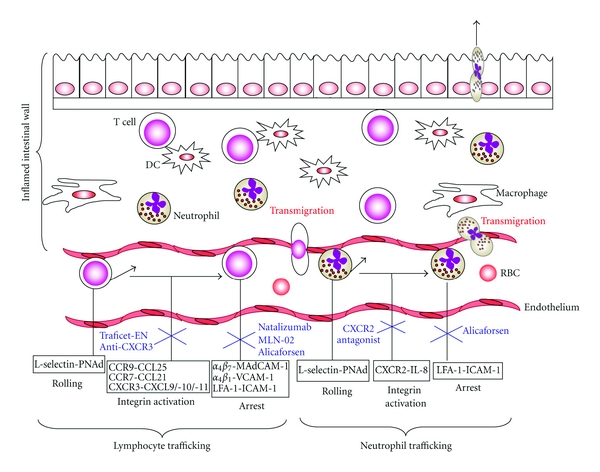
Schematic view of the leukocyte trafficking adhesion cascade in IBD. Leukocytes tether and roll along the vascular endothelium via selectin-mediated adhesion. They are then activated by chemokines into a high avidity, high affinity state so that integrin-mediated strong adhesion and arrest can take place. This prepares the leukocyte for transmigration through the blood vessel wall into the inflamed colon. Chemokine activation can be inhibited by various chemokine/chemokine receptor inhibitors such as the CCR9 small molecule antagonist Traficet-EN, a monoclonal antibody to CXCR3 or a CXCR2 antagonist (shown in blue). Additionally, antagonists of integrin firm adhesion include the anti- *α*_4_ integrin monoclonal antibody Natalizumab, the selective *α*_4_ *β*_7_ small molecule antagonist MLN-02 and the antisense intercellular adhesion molecule-1 (ICAM-1) oligonucleotide Alicaforsen (shown in blue). KO, knock out; LFA-1, Lymphocyte function-associated antigen 1; MadCAM-1, mucosal addressin-cell adhesion molecule 1; PNAd, Peripheral lymph node addressin; RBC, red blood cell; V-CAM-1, vascular-cell adhesion molecule 1.

Circulating leukocytes are subjected to extreme conditions with the flow of blood exerting a shearing stress on the cells dislodging any that touch the vascular wall. To leave the circulation and home to specific tissues, leukocytes must engage several adhesion pathways involving intimate interaction with endothelial cells [26]. Whether during physiological recirculation or inflammatory conditions, the mechanisms involved in leukocyte trafficking are effectively the same. The leukocyte trafficking cascade is depicted in Figure 1. Leukocyte recruitment begins with tethering and rolling of the cells along the microvascular endothelium via three selectins: L-selectin, expressed by leukocytes, and E-selectin and P-selectin, expressed by inflamed endothelial cells on the blood vessel wall [27]. The ligand P-selectin glycoprotein ligand-1 (PSGL-1) binds all three selectins and plays an important role in leukocyte recruitment under inflammatory conditions [28]. L-selectin is constitutively present on T cells and interacts with its counter receptors, peripheral lymph node addressin (PNAd) and mucosal addressin cell adhesion molecule (MAdCAM)-1, acting as a mechanical anchor or tether to the high endothelial venules (HEVs). This allows the lymphocytes to roll along the vascular lining at a much slower pace than erythrocytes. Selectins engage rapidly and form strong bonds to secure contact. These bonds allow chemokines and their ligands to transmit activating signals for the next step in the migration cascade. Targeting selectins and their ligands as a treatment strategy for inflammatory disorders has been reviewed elsewhere [29].

Selectin bonds are unable to arrest cells at the vessel wall. Firm leukocyte adhesion is achieved through bonds formed downstream by the secondary adhesion molecules, integrins. Integrins are diversely expressed on different leukocyte subpopulations and are composed of noncovalently linked *α* and *β* chains. The *α*_4_ integrins, *α*_4_ *β*_1_ and *α*_4_ *β*_7,_ play a regulatory role in lymphocyte homing and recruitment to inflammatory tissues, particularly to the inflamed intestine. Two decades ago, it was revealed that memory T cells from the gut preferentially homed to the gut [30]. This phenomenon is linked to the expression of unique adhesion molecules within the mucosa [31, 32]. Both *α*_4_ *β*_1_ and *α*_4_ *β*_7_ are expressed by lymphocytes that reside in the gut and gut-associated lymphoid tissues (GALTs), and their respective ligands vascular cell adhesion molecule (VCAM)-1 and MAdCAM-1 are expressed within the HEV's in Peyer's patches (PPs) and the flat-welled venules of the lamina propria [33]. While the *α*_4_ *β*_7 _-MAdCAM-1 interaction is restricted to leukocyte trafficking to the gut and GALT, the *α*_4_ *β*_1_-VCAM-1 pathway can also mediate leukocyte homing to the central nervous system, specifically to the inflamed brain [34]. Interestingly, mice deficient in the *β*_7_ integrin gene are unable to form proper PPs and possess decreased numbers of lamina propria CD4^+^ T cells and B cells [35]. The *β*_2_ integrins are also prominent participants in leukocyte trafficking, mediating firm adhesion, particularly in the case of neutrophils [36]. The *β*_2_ integrin lymphocyte function-associated antigen (LFA)-1 is predominantly expressed by lymphocytes and neutrophils and binds to its endothelial cell ligands intercellular adhesion molecule (ICAM)-1 and ICAM-2. In addition to leukocyte arrest, integrins can participate in leukocyte rolling. Under inflammatory conditions, lymphocytes can skip the selectin-mediated phase and bind directly to endothelial cells via *α*_4_ *β*_7_ [37].

Abundant evidence reveals that IBD is associated with enhanced leukocyte trafficking to the gut mucosa and altered expression of adhesion molecules [38]. Cytokines such as interleukin (IL)-1*β*, IL-6, and TNF-*α*, produced upon stimulation of innate immune cells at inflammatory sites [39], upregulate adhesion molecules and chemokines, enhance leukocyte recruitment, and amplify the inflammatory cascade. Expression of MAdCAM-1 is upregulated in animal models of colitis [40, 41] and in active IBD [42–44]. ICAM-1 and LFA-1 have also been implicated in a number of experimental animal models of IBD [45, 46]. The importance of adhesion molecules in IBD is evident from preclinical colitis studies, where their blockade ameliorated disease severity [47].  Table 1 summarizes preclinical and clinical data reported so far. Similar results have been reported in animal models of autoimmune disease including MS and rheumatoid arthritis [48].

#### 3.2.2. Chemokines and Their Receptors

Chemokines mediate cell migration under normal physiological conditions and leukocyte recruitment to tissues during innate and adaptive immune responses. These small heparin-binding proteins come from a diverse family that is classified into four major subfamilies, CC, CXC, C, and CX_3_C, based on structural and functional differences. The two most important subgroups in terms of leukocyte trafficking to inflamed tissues are the CC chemokines for dendritic cell (DC) and lymphocyte recruitment and the CXC chemokines for recruitment of neutrophils and monocytes. Chemokines exert their biological effects on target cells by binding to specific G-protein-coupled transmembrane receptors (GPCRs) on the cell surface and activating an intracellular signaling cascade. Consequently, an activating signal is sent to the integrin switching it into a high-affinity/high-avidity state so that the rolling leukocyte can arrest itself and firmly adhere to the HEVs, a step which is essential for leukocyte extravasation into the target tissue [74]. For example, binding of CCR7 on naïve T cells to its chemokine ligand CCL21 on HEVs in turn activates binding of *α*_4_ *β*_7_ and LFA-1 to their endothelial ligands MAdCAM-1 and ICAM-1, respectively [38]. In addition, the combined expression of chemokine receptors and adhesion molecules by naïve and memory T lymphocytes govern their selective homing patterns. For instance, while L-selectin and CCR7 regulate naïve T-cell migration to peripheral LNs, expression of *α*_4_ *β*_7_ in conjunction with CCR9 allows T-cell migration to the skin and gut. The encounter between CCR7 and its ligands CCL19 and CCL21 bridges the gap between innate and adaptive immune responses. Up-regulation of CCR7 on antigen-laden DCs facilitates their migration into LNs for T-cell priming. In addition, enhanced expression and binding of CCR7 on naïve T cells to CCL19 on DCs and CCL21 on HEVs mediate transmigration from peripheral tissues into LNs. In contrast, downregulation of CCR7 allows activated T cells to exit the LN area and migrate to target tissues to carry out effector functions [75, 76]. Furthermore, elevated expression of CXCR5, the ligand of CXCL13, on certain CD4^+^ T cells directs their migration to the follicle to provide B-cell help [77]. Upregulated mucosal expression of numerous chemokines and their counter receptors including CXCL8 (IL-8)/CXCR2, CXCL9,10,11/CXCR3, CCL25/CCR9, CCL19,21/CCR7, and CCL20/CCR6 is evident in active IBD and in models of colitis [78–80]. Thus, chemokines orchestrate the activation, recruitment, and retention of leukocytes, and, the more insight we gain into their vital role, the more attractive they become as potential therapeutic targets.

#### 3.2.3. The Role of DCs in Lymphocyte Homing to the Gut

The specific homing receptors expressed by activated T cells are determined by DCs. Several murine studies show that DCs from mesenteric lymph nodes (MLNs) or PPs imprint gut tropism on antigen-experienced T cells, by inducing expression of the gut homing receptors *α*_4_ *β*_7_ and CCR9 [81–83]. In the “steady state” mouse intestine, the ability to confer gut homing specificity is restricted to the CD103^+^ intestinal DC subset [84–86]. The vitamin A metabolite retinoic acid (RA) plays a central role in the process of DC imprinting within lymphoid tissues. For instance, mice deficient in vitamin A have decreased numbers of T cells in the intestine [37, 87, 88]. However, the dependency of DC-T-cell imprinting on retinoic acid receptor (RAR) signaling is complex and not yet fully characterized [89]. Also, the key factors that induce DC imprinting activity have yet to be identified; for example, germ-free studies have indicated that gut bacteria are not required for intestinal DC imprinting of *α*_4_ *β*_7_ expression [90]. The specific role played by intestinal DC imprinting in IBD remains elusive, but presumably it influences the increased numbers of *α*_4_ *β*_7_^+^ and CCR9^+^ lymphocytes evident in the inflamed intestine [91]. Since the CD103^+^ intestinal DC subset favors the generation of Foxp3^+^ T-regulatory cells (Tregs) over Th17 cells, via a TGF-*β*- and RA-dependent mechanism [92], targeting leukocyte trafficking at the DC imprinting level may represent a potential therapeutic strategy for IBD. Notably, a diet low in vitamin A protects against colitis in mice, and this protection is associated with increased levels of Tregs in the gut mucosa. In this case, the reduced availability of RAR ligands affects lymphocyte homing to the gut by decreasing entry of *α*_4_ *β*_7_ and CCR9^+^ T cells in favor of Tregs [93].

#### 3.2.4. S1P and Control of Leukocyte Egress from Tissues

While chemokines control naïve T-cell migration to and within LNs, the natural bioactive lipid sphingosine-1-phosphate (S1P) regulates lymphocyte egress. S1P is formed upon the phosphorylation of sphingosine by sphingosine kinase and mediates a number of fundamental biological events including endothelial barrier enhancement, lymphocyte differentiation and immune cell trafficking [94]. Levels of S1P in the blood and lymph are constitutively high while they are low in tissues, increasing substantially upon inflammation. Although synthesized in most cells, tissue levels of S1P are tightly controlled due to intracellular degradation by S1P lyase or phosphorylation by S1P phosphatases. Five S1P receptors have been described so far, S1PR1–5, and are expressed in a cell-type specific manner within different tissues [95]. S1PR1–3 are ubiquitously expressed in mammals [96], while S1PR4 expression is restricted to lymphoid tissues [97] and lung, and S1PR5 to brain, skin, and natural killer (NK) cells [98]. The S1P/S1PR1 axis is essential for lymphocyte egress from the thymus and spleen into the blood and from the LNs into the lymph [99]. During T-cell priming, elevated expression of CD69 on activated lymphocytes promotes the temporary downregulation of S1PR1 expression on the cell membrane, disabling ligation to S1P and consequently trapping them within the LNs [100]. Concurrent binding of CCR7 to its ligand CCL21 mediates competitive retention signals. Following clonal expansion, CCR7 expression is lost and S1PR1 expression is upregulated once again, allowing the effector T cells to leave the lymphoid tissues, reenter the systemic circulation, and rapidly migrate to sites of inflammation [101]. The S1P/S1PR interaction also regulates the movement of DCs [102], neutrophils [103], and NK cells [104]. S1P signaling and functions in immunity have been reviewed elsewhere [105].

The discovery that the S1P/S1PR1 axis is essential for lymphocyte egress added a new potential target for blocking leukocyte migration in inflammatory diseases. Disruption of this S1P gradient has been reported in numerous inflammatory and/or autoimmune disorders including asthma [106] and rheumatoid arthritis [107]. The novel immunosuppressant FTY720 (Fingolimod) [108] is structurally similar to S1P and poses as an S1P analog. Like S1P, it is phosphorylated *in vivo* and binds with high affinity to 4 of the 5 S1P receptors S1PR1, S1PR3, S1PR4, and S1PR5 [109]. FTY720 interferes with S1P signaling and blocks the response of lymphocytes to egress signals from the lymphoid organs, sequestering them within the LNs and PPs. The result is a rapid and dramatic peripheral blood lymphopenia with depletion of circulating T and B cells. In contrast, FTY720 increases the number of DCs in the blood and simultaneously reduces their numbers in secondary lymphoid organs. In addition, it can modulate DC cytokine signaling potentially affecting T-cell responses [110]. The mechanism of action of FTY720 is complex, and it is currently unclear whether it acts as an agonist or functional antagonist or both during regulation of lymphocyte recirculation *in vivo*. Since FTY720 inhibits cell migration to inflammatory sites, it has shown great potential as a treatment for inflammatory disorders [108]. In clinical studies, FTY720 successfully prevented kidney transplant rejection [111–114] and proved highly effective in treating MS. It was recently approved by the US Food and Drug Administration (FDA) as a first-line treatment for relapsing forms of MS. In terms of IBD, FTY720 ameliorated experimental colitis arising as a result of chemical induction [115–117], T-cell transfer [118], and IL-10 deficiency [119], suggesting it may be a potential candidate for IBD treatment. However, reports of side effects such as bradycardia and increased susceptibility to opportunistic infections [108] dictate that the use of FTY720 therapeutically should be approached with caution and that perhaps using more selective drugs to target the S1P receptor pathway may be a safer option and yield less side effects. Indeed, the specific agonist of S1PR1, SEW2871, has shown promise in preclinical kidney transplantation studies [120] and exhibited anti-inflammatory effects in mice administered TNF-*α* [121]. In addition, another selective S1PR1 agonist KRP-203 showed therapeutic potential in IL-10-deficient mice [122].

## 4. Preclinical and Clinical Evidence of Targeting Adhesion Molecules

Figure 1 and Table 1 summarize the data presented under Sections 4 and 5.

### 4.1. Natalizumab

Promising preclinical data [22, 49, 50, 54, 55, 57, 61, 62] (also see Table 1) led to the use of humanized *α*_4_ integrin antibodies in clinical trials. The most well-known anti-*α*_4_ drug is Natalizumab (Tysabri), a humanized pan-*α*_4_ monoclonal antibody. Natalizumab blocks the ability of *α*_4_ *β*_1_ and *α*_4_ *β*_7_ to bind to their respective ligands on the endothelium, preventing lymphocyte transendothelial migration. This *α*_4_ antagonist was approved by the FDA in 2004 and is highly effective in treating the symptoms of MS [123, 124] and in preventing relapse and increasing remission rates in sufferers with moderate to severe CD [51, 52]. Natalizumab therapy has been associated with cases of progressive multifocal leukoencephalopathy (PML) in a small number of patients, which is induced by the JC virus, an opportunistic infection of the brain [125]. Though rare, PML is a serious and often fatal disease. The approval and relative success of Natalizumab have heightened interest and greatly encouraged further research into targeting integrins and their counter adhesion molecule ligands as a novel treatment strategy for chronic inflammatory diseases, including IBD.

### 4.2. MLN-02

Vedolizumab (MLN-02) is a recombinant humanized IgG1 monoclonal antibody selective for the gut-specific integrin *α*_4_ *β*_7_. By binding to *α*_4_ *β*_7_, MLN-02 inhibits the adhesion and migration of leukocytes into the gastrointestinal tract, preventing intestinal inflammation. MLN-02 treatment ameliorated disease in the cotton top tamarin model of colitis [126] and safely and effectively induced clinical response and remission in two double-blinded placebo-controlled clinical trials of patients with active CD and UC [58–60]. The selectivity of MLN-02 makes it less likely to impair systemic immunity and more attractive as a therapeutic target for IBD.

### 4.3. Alicaforsen- (ISIS2302-) and LFA-1-Targeting Drugs

Alicaforsen, a human ICAM-1 antisense oligonucleotide, inhibits ICAM-1 production preventing T-cell adhesion, extravasation, and subsequent migration to inflamed areas [127]. Blocking ICAM-1 ameliorated colitis in a number of preclinical models [63, 64, 67]. In clinical studies, this approach has had variable success and in general has yielded disappointing results in the treatment of CD [128–130]. However, there have been promising results with an enema formulation of Alicaforsen in the treatment of UC [65] and refractory pouchitis [131]. Efalizumab, a humanized monoclonal IgG1 antibody treatment for plaque psoriasis, is FDA approved and also acts by blocking the LFA-1/ICAM-1 interaction. By doing this it inhibits T-cell migration to the inflamed dermal and epidermal tissues. However, similar to Natalizumab, serious adverse effects, such as the Epstein-Barr virus-associated B-cell lymphoma development, were reported following treatment [132].

### 4.4. Small Molecule Antagonists

**Figure 2:**
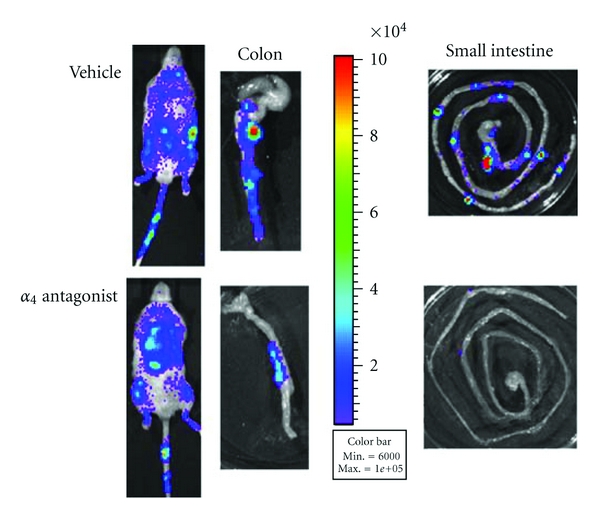
Inhibition of leukocyte migration by an *α*_4_ integrin antagonist in experimental colitis. Leukocytes were isolated from mesenteric lymph nodes (MLNs) of *β*-actin luciferase mice and injected into recipient mice with dextran sodium sulphate- (DSS-) induced colitis. The recipient mice received vehicle or *α*_4_ antagonist, 1 hour pre cell transfer. Whole body and organs *ex vivo* (colon and small intestine) were imaged using an IVIS 100 charge-coupled device imaging system 4 hours following transfer. The pseudocolored images represent light intensity, where red is the strongest, and violet is the weakest signal. Inhibition is detected in the colon and in Peyer's patches of the *α*_4_ antagonist-treated mice.

The immunogenicity of antibody therapies has increased research into the use of nonpeptide small molecule antagonists to block leukocyte trafficking. Such therapeutics are less likely to elicit the undesirable and serious immunogenic responses associated with monoclonal antibody therapy, and, unlike antibodies, they can be taken orally and are less expensive to produce. We analyzed the leukocyte trafficking blockade effect of a small molecule *α*4 integrin antagonist in a preclinical model of IBD. We confirmed the therapeutic efficacy of the compound in dextran sodium sulphate- (DSS-) induced acute colitis and demonstrated its ability to inhibit leukocyte trafficking to the inflamed gastrointestinal tract *in vivo* using bioluminescence imaging, as shown in Figure 2 [22]. Previous studies using small molecule integrin antagonists in other models of inflammatory disease have also shown promising results [133, 134]. 

## 5. Preclinical and Clinical Evidence of Targeting Chemokines

### 5.1. CCR9

The chemokine CCR9 is exclusively expressed by gut homing leukocytes and interaction with its counter ligand CCL25 is essential for T-cell homing to the small intestine [135]. The CCR9/CCL25 interaction specifically contributes to the pathophysiology of small bowel CD [136]. Antibody blockade of this interaction reduced inflammation in early stages of chronic ileitis in senescence accelerated (SAMP1/Yit) mice [68]. Additionally, pre- or post administration of a small molecule CCR9 antagonist (CCX282-B/Traficet-EN) reduced gut inflammation in TNFΔARE mice, an experimental model of CD [69]. Interestingly, Wermers et al. recently demonstrated that blockade of CCR9 exacerbated chronic ileitis in these mice, by inhibiting recruitment of Tregs to the small intestinal lamina propria and MLNs [137]. The exact role of CCR9/CCL25 in large intestinal inflammation remains unclear, and studies have yielded conflicting results. Preliminary clinical data using Traficet-EN demonstrated a beneficial therapeutic effect in both patients with ileal and colonic CD, by significantly reducing proinflammatory cytokine levels and disease scores and maintaining clinical remission [70]. This is surprising, since there is little or no expression of CCL25 in the colon [138, 139]. However, recent data showed that, although colonic levels of CCL25 are low in healthy mice, they are significantly upregulated upon DSS-induced colitis. In addition, CCR9 knock-out (KO) mice with acute DSS colitis exhibit enhanced severity of clinical symptoms and tissue injury and display delayed recovery. Exacerbation of disease was associated with an imbalance in DC subpopulations and increased macrophage infiltration into the colon [138]. These data suggest that use of CCR9 blockade therapy in, for example, strictly colonic UC, could have detrimental effects.

### 5.2. CXCR3

CXCR3 is expressed by monocytes, T cells, and NK cells and can mediate their recruitment to inflammatory sites by binding to its ligands CXCL9, CXCL10, and CXCL11. CXCR3 engagement with these chemokines mediates the rapid arrest of effector T cells *in vitro* [140] and selectively mobilizes high-CXCR3-expressing Th1 cells to sites of mucosal inflammation [141]. Expression of CXCR3 and its chemokine ligands is elevated in both preclinical and clinical models of IBD [142]. CXCL10 is considered the most crucial and potent chemokine in CXCR3-mediated chemotaxis, as it is highly upregulated and its expression robustly correlates with disease severity in inflammatory disorders such as IBD, MS, and arthritis [143–145]. Moreover, the ability of CXCL10 to preferentially attract Th1 cells emphasizes its contribution to these diseases [146]. CXCL10 antagonism prevented or ameliorated inflammation in numerous preclinical models of inflammatory disease [142]. More specifically, neutralization of CXCL10 using monoclonal antibody therapy proved effective in various experimental models of IBD [71–73]. In contrast, in a more recent study, though antibody blockade of CXCL10 reduced intestinal epithelial cell proliferation and CXCR3^+^ cell migration *in vitro *and *in vivo*, it had no significant effect on disease in several preclinical models including IBD, arthritis, and MS [147]. The reasons for the discrepancies between these studies are unclear, but differing methods of antagonism and disease induction may play a part. For instance, Byrne et al. used T-cell (CD4^+^CD45RB^Hi^) transfer to induce colitis, while earlier studies employed the IL-10KO and DSS-induced colitis models. Future clinical trials are likely to resolve these uncertainties (NCT01294410).

### 5.3. CXCR2

Since neutrophil influx into the intestinal mucosa and resulting tissue damage is a major characteristic of active IBD, especially UC, neutrophil-specific chemokine receptors, and their ligands also represent potential therapeutics. Neutrophils exclusively use integrins of the *β*_2_ family to arrest on the endothelium and antibodies against these integrins reduced tissue damage in experimental colitis [148]. Engagement of the chemokines human IL-8/CXCL8 and the murine functional homologs CXCL1 and CXCL2 with their receptors CXCR1 and CXCR2 triggers numerous signal transduction cascades, which in turn activate neutrophil recruitment to target tissues [149]. Inflammatory mediators such as bacterial lipopolysaccharide (LPS), TNF-*α*, and IL-1 stimulate the production of IL-8. Upregulation of these chemokines in conjunction with polymorphonuclear cells (PMN) infiltration into the inflamed intestinal mucosa correlates well with the degree of active inflammation and tissue injury in human and experimental models of IBD [78, 150, 151]. CXCR2 is a well-established mediator of PMN recruitment in preclinical models of inflammatory disease [152–154]. A small molecule CXCR2 antagonist (SB225002) was effective in ameliorating trinitrobenzene sulfonic acid- (TNBS-) induced colitis in mice, as was an anti-CXCL1 antibody [155]. We used bioluminescence imaging of adoptively transferred luciferase-expressing neutrophils to study the kinetics of neutrophil migration in acute DSS-induced colitis. This enabled demonstration of preferential recruitment of the neutrophils to the inflamed colon and the blockade effect of an anti-CXCL1 antibody on the trafficking neutrophils [21].

### 5.4. The “Redundancy” Issue

The promise of drug inhibition of chemokines and their receptors in IBD has not yet been realized. The chemokine system is “redundant,” and the same biological function can be carried out by several chemokines and their receptors *in vivo*. This questions their suitability as anti-inflammatory drug targets. However, it was recently pointed out that the lack of progress in chemokine strategies may not be due to “redundancy,” but rather to the shortcomings in the approaches employed to target them [156]. Clinical efficacy of a small molecule CCR9 antagonist was demonstrated in patients with moderate to severe CD [157], proving that targeting one chemokine receptor can be therapeutically successful in the treatment of chronic inflammation.

## 6. Future Directions: MicroRNAs

Recent studies suggest that microRNAs (miRNAs) play a specific role in the posttranscriptional regulation of leukocyte trafficking. miRNAs are small (21−23 nucleotide) noncoding RNAs that control gene expression. By targeting complementary messenger RNAs (mRNAs) for degradation or translational repression, they suppress the expression of protein-coding genes. Blockade of miR-126 function using an antagomir, a single-stranded antisense-like molecule, suppressed the Th2 response and subsequently the development of disease in an experimental model of allergic asthma [158]. Interestingly, miR-126 was identified as one of a set of miRNAs expressed in endothelial cells [159, 160] and was shown to inhibit TNF-induced endothelial expression of VCAM-1, thus blocking leukocyte adhesion via its lymphocyte integrin ligand *α*_4_ *β*_1_ [161]. In terms of neutrophil trafficking, downregulation of E-selectin and ICAM-1 by miR-31 and miR-17-3, respectively, controlled neutrophil binding to endothelial cells [162]. Inhibition of these miRNAs using specific antagonists increased neutrophil adhesion to endothelial cells *in vitro, *while transfecting with mimics (agonists) of these miRNAs had the opposite effect. This study suggests that miRNAs negatively regulate inflammatory processes. In another study, miR-7 downregulated expression of CD98, a lymphocyte receptor that regulates integrin signaling [163]. CD98 levels were increased in the inflamed colons of patients with CD, while miR-7 levels were decreased. Taken together, the biological importance of miRNAs in the pathogenesis of IBD is becoming clearer, and targeting miRNAs in the context of leukocyte trafficking may be a safer approach for future therapeutic opportunities.

## 7. Concluding Remarks

Targeting leukocyte migration is a realistic strategy, and Natalizumab shows proof of principle. Compounds inhibiting a single chemokine, such as CCR9, have also shown promise, but awareness is necessary. Targeting molecules such as miRNAs, histone deacetylases [164], or the family of bromodomain proteins [165], which regulate gene expression programs that govern endothelial cell function in inflammatory settings, may represent a new generation of drugs for IBD.

## Figures and Tables

**Table 1:** Targeting leukocyte trafficking in inflammatory bowel disease.

Target	Drug type	Preclinical efficacy	Therapeutic	Clinical efficacy	
*Adhesion molecules *					
*α*_4_ Integrins/ligands	Antisense MAdCAM-1 oligonucleotide	TNBS colitis [49, 50]	Natalizumab (humanized IgG4 mAb anti-*α*_4_ integrin)	CD (Phase IV) [51, 52]	Prevented relapse, induced remission
	Anti-VCAM-1 mAb			UC [53]	Pilot study
	Anti-MAdCAM-1 mAb	DSS colitis [54, 55]	AJM300 (orally available anti-*α*_4_ integrin mAB)	CD (Phase II) [56]	Reduced disease activity, good safety profile
	Small molecule *α*_4_ integrin antagonist	DSS colitis [22]			
	Anti-*β*_7_ and anti-MAdCAM-1 mAb	T-cell transfer colitis [57]	Vedolizumab/MLN-02 (humanized IgG4 mAb *α*_4_ *β*_7_ integrin)	CD [58], UC Phase II [59, 60]	Induced clinical response and remission, good safety profile
	Anti-MAdCAM-1 mAb	SAMP1/Yit mice [61]			
	Anti-*α*_4_ *β*_7_ mAb	Cotton top tamarin model [62]			

ICAM-1/LFA-1	Anti-ICAM-1 mAb Antisense ICAM-1 oligonucleotide	DSS colitis [63, 64]	Alicaforsen (ISIS2303) (antisense ICAM-1 oligonucleotide)	UC (Phase II) [65, 66]	Reduced disease activity, good safety profile
	Anti-ICAM-1 mAb	SAMP1/Yit mice [67]			

*Chemokines *					
CCR9/CCL25	Anti-CCR9/CCL25 mAb Traficet-EN (CCX282-B)	SAMP1/Yit mice [68] TNF (DeltaARE) mice [69]	Traficet-EN/CCX282-B (small molecule CCR9 antagonist)	CD (Phase III) [70]	Induced clinical remission, good safety profile
CXCR3/CXCL10	Anti-CXCL10 mAb	IL-10 KO [71, 72] DSS colitis [73]	MDX-1100 (humanized anti-CXCL10 mAb)	UC (Phase II)	NCT00295282 NCT00656890

CD: Crohn's disease; DSS: dextran sodium sulphate; ICAM-1: intercellular adhesion molecule 1; MadCAM: mucosal addressin-cell adhesion molecule 1; senescence accelerated mice (SAMP1/Yit); TNBS: trinitrobenzene sulfonic acid; UC: ulcerative colitis; V-CAM-1: vascular-cell adhesion molecule 1.

## Abbreviations

BLI: Bioluminescent imaging
CD: Crohn's disease
DC: Dendritic cell
DSS: Dextran sodium sulphate
GALT: Gut-associated lymphoid tissues
GPCRs: G-protein-coupled transmembrane receptors
HEV: High endothelial venules
ICAM-1: Intercellular adhesion molecule 1
IBD: Inflammatory bowel disease
IL: Interleukin
KO: Knock out
LFA-1: Lymphocyte-function-associated antigen 1
LN: Lymph node
LPS: Lipopolysaccharide
MadCAM: Mucosal addressin-cell adhesion molecule 1
MLNs: Mesenteric lymph nodes
MPO: Myeloperoxidase
MRI: Magnetic resonance imaging
mRNA: Messenger RNA
miRNAs: MicroRNAs
MS: Multiple sclerosis
NK: Natural killer
PET: Positron emission tomography
PMN: Polymorphonuclear cells
PNAd: Peripheral lymph node addressin
PPs: Peyer's patches
PSGL-1: P-selectin glycoprotein ligand 1
RA: Retinoic acid
RAR: Retinoic acid receptor
SPECT: Single photon emission computed tomography
S1P: Sphingosine-1-phosphate
TNF-*α*: Tumor necrosis factor-*α*
TNBS: Trinitrobenzene sulfonic acid
Tregs: T-regulatory cells
UC: Ulcerative colitis
V-CAM-1: Vascular-cell adhesion molecule 1.
